# Living alone and mortality risk in cancer patients: a systematic review and meta-analysis

**DOI:** 10.3389/fonc.2026.1866474

**Published:** 2026-08-11

**Authors:** Jinrong Huang, Zuolang Tu, Daolin Wu, Fuwei Liu, Lihui Du

**Affiliations:** 1 Department of Sleep Medicine, The Affiliated Ganzhou Hospital, Jiangxi Medical College, Nanchang University, Ganzhou, Jiangxi, China; 2 Department of Cardiology, The Affiliated Ganzhou Hospital, Jiangxi Medical College, Nanchang University, Ganzhou, Jiangxi, China

**Keywords:** All-cause mortality, cancer, living alone, meta-analysis, systematic review

## Abstract

**Background:**

Living alone has become increasingly prevalent across the globe, but its association with all-cause mortality among cancer patients remains unclear. This systematic review and meta-analysis evaluated the relationship between living alone and all-cause mortality risk in cancer patients.

**Methods:**

A comprehensive search of PubMed, Embase, Cochrane Library, and Ovid Medline was performed up to July 10, 2026. We included cohort studies that reported adjusted hazard ratios (HRs) and 95% confidence intervals (CIs) for all-cause mortality comparing cancer patients living alone with those not living alone. Pooled estimates were calculated using an inverse-variance random-effects model.

**Results:**

Twenty-one cohort studies comprising 2,497,397 cancer patients (55.6% female) were included. Compared with patients not living alone, those living alone had a significantly higher risk of all-cause mortality (HR = 1.22, 95% CI: 1.13–1.32). In sex-specific analyses, the pooled HR was 1.34 (95% CI: 1.23–1.45) for men and 1.15(95% CI: 1.00–1.32) for women, with no statistically significant subgroup difference (p = 0.06), indicating no clear sex difference in this association.

**Conclusion:**

Living alone is associated with increased all-cause mortality in cancer patients, with no apparent sex difference.

**Systematic Review Registration:**

https://inplasy.com/inplasy-2024-10-0051/, identifier INPLASY2024100051.

## Introduction

Living alone has become increasingly common as the population ages and birth rates decline. Studies have shown that in 2017, one-third (33.6%) of households in the European Union (EU) and about 40% of households in the Nordic countries (except Iceland) were single-occupancy households (1). Globally, the number of people living alone among older adults and working adults is likely to continue increasing (2, 3). Definitions of living alone may vary. Formal marital status no longer necessarily reflects a person’s living arrangements, as single, divorced and widowed people may live alone or with others, such as partners, children, parents or other unrelated persons. Thus, living arrangements may be more indicative of a person’s social bonds than formal marital status. In addition, people living alone are not a homogeneous group. People living alone may be at different stages of life, depending on age, sex, education and work status. Moreover, living arrangements may change several times during a person’s lifetime. Living alone is understood as having only one person living in a household at the time of the study, in other words, a household with only one person (3). Previous research has suggested that living alone may adversely affect mental health and quality of life, particularly among older adults (4, 5). Some studies have found that living alone is associated with an increased risk of premature death, cardiovascular disease, stroke, and overall poor physical health (6, 7). In addition, living alone has been associated with a poorer prognosis in patients with diabetes, heart failure and coronary artery disease (8–10). The above studies suggest that living alone may be becoming a detrimental factor to social and public health.

Cancer is a major public health problem that requires global attention. It is predicted that the number of cancer cases will increase by 68%, reaching 23.6 million cases per year by 2030 (11, 12). This rising trend will place a greater burden on society. The incidence of cancer is closely related to age, and it is predicted that by 2040, 30 million new cases of cancer will be diagnosed each year, with almost 80% of these cases occurring in adults over the age of 50 (13). There is limited research on the impact of living alone as a lifestyle on cancer in this particular population. Recent studies have shown that cancer patients living alone face a higher risk of death compared to cancer patients with social support (14, 15). A recent meta-analysis found that living alone was associated with increased total mortality compared with non-living alone adults, with younger people at higher risk than older people and men at higher risk than women (16). However, the potential relationship between living alone and the risk of all-cause mortality in cancer patients has not been clarified, nor is it known whether there are sex differences in this risk. Therefore, we aimed to explore the relationship between living alone and the risk of all-cause mortality from cancer.

## Methods

This investigation adhered to the PRISMA (Preferred Reporting Items for Systematic Reviews and Meta-Analyses) statement (17).The study was registered on the INPLASY website (registration number: INPLASY2024100051).

### Literature search

A comprehensive search was conducted in electronic databases including PubMed, Embase, Cochrane Library, and Ovid Medline up until July 10, 2026. The MeSH terms and free text terms were (“living alone”) AND (“tumor” OR “neoplasms” OR “malignancy” OR “cancer”). Language restrictions were not imposed during the literature search. Additionally, the bibliographies of the retrieved studies were examined to identify any additional reports and ensure the thoroughness of the literature search.

### Study selection

Two researchers independently completed the entire process, including literature search, selection, and data analysis. We used Endnote X9 software (Thomson Reuters, New York, NY, USA) to manage the research data. After removing duplicate studies automatically and manually, we initially screened the relevant literature by examining titles and abstracts. If articles or other information were not available, we contacted the corresponding authors to acquire the required data. Subsequently, we thoroughly reviewed the initially screened literature to identify the final set of eligible studies. Any discrepancies that emerged during this process were resolved by the third reviewer.

The studies were selected based on the PICOS principles, with the following inclusion criteria: (1) Participants included in the studies were patients with cancer. (2) The exposure and comparator variables were Living alone and non-living alone, respectively. (3) The outcome of interest was mortality. (4) The included studies were observational in nature and published as full-length articles. (5) The effect estimates included adjusted hazard ratios (HRs) and 95% confidence intervals (CIs). Additionally, studies were excluded if they were reviews, meta-analyses, purely abstract articles, or focused on other outcomes. Furthermore, studies were excluded if data could not be extracted or were not reported.

### Data extraction and quality assessment

Two review authors independently extracted relevant information from eligible studies, and any discrepancies were resolved through consensus. Data extraction was performed using pre-designed Microsoft Excel 2019 (Microsoft Corporation, Redmond, WA, USA), and the following information was extracted from the studies: (1) First author’s name; (2) Year of publication; (3) Country or region; (4) Study type; (5) Follow-up duration; (6) Basic characteristics (sample size, mean age, proportion of women); (7) Participant source; (8) Study results; (9) Adjustment for confounders; and (10) Hazard ratios (HR) and corresponding 95% confidence intervals (CIs) extracted from the model with the most adjustments. For the included observational cohort studies, we used the Newcastle-Ottawa Scale (NOS) to assess risk of bias (18). The inter-rater agreement for literature screening was assessed using Cohen’s kappa statistic, with a kappa value of 0.78 (95%CI 0.66–0.87), indicating substantial agreement between the two reviewers.

### Statistical analysis

Review Manager 5.3 software was used to carry out all statistical analyses for this study. The two most often utilized statistical techniques to measure heterogeneity were the Cochrane Q test and the I^2^ statistic, with P < 0.1 or I^2^ > 50%, respectively, indicating significant heterogeneity. An inverse variance random effects model was then used to pool the natural logarithms of the HRs and standard errors of the included studies.

## Results

### Literature search

**Figure 1:**
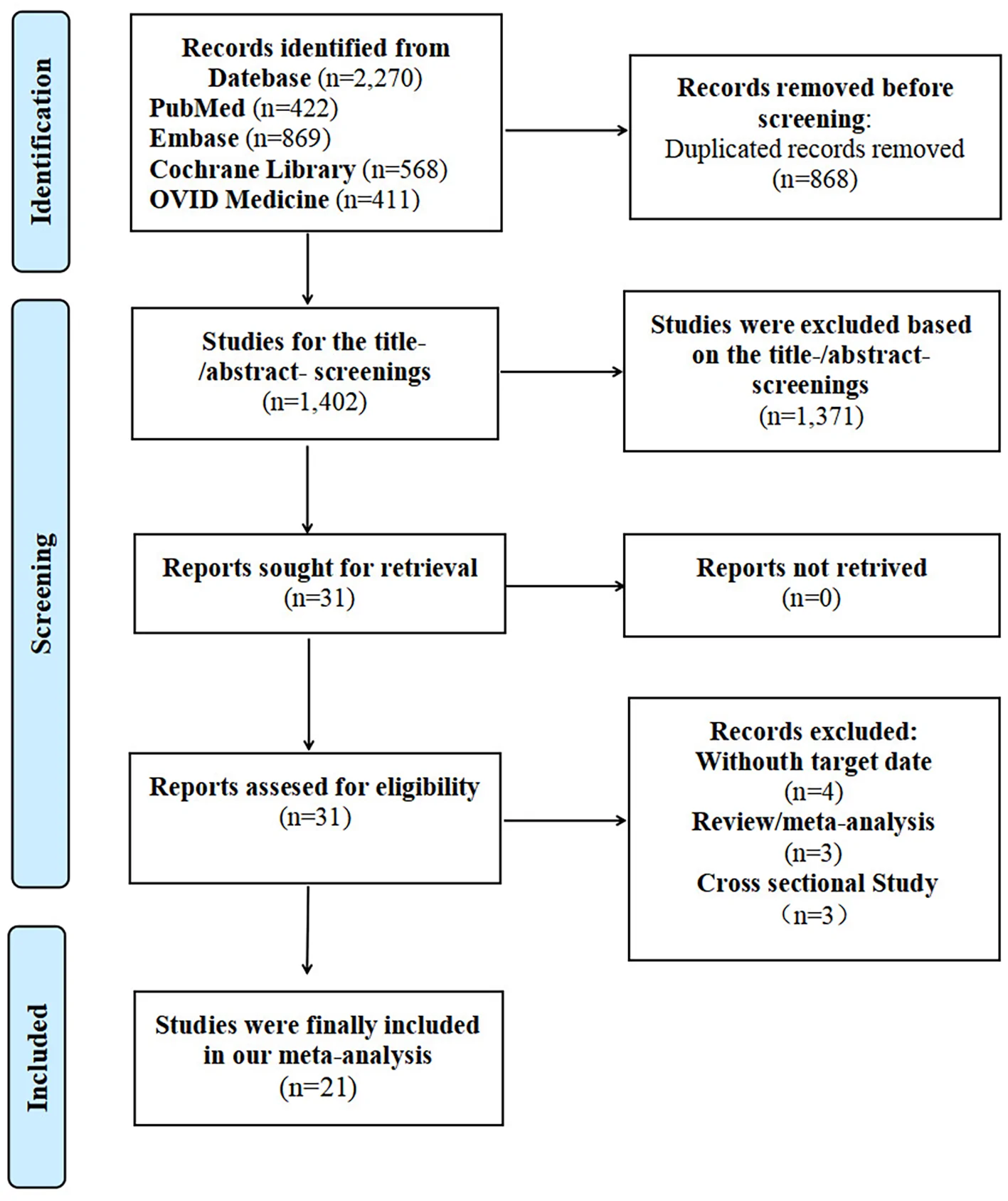
The process of the literature retrieval of this meta-analysis.

Using a predefined search strategy (**Supplementary Table 1**), we retrieved 2,270 studies from the database. After removing duplicates, we excluded 868 studies. We then screened titles, abstracts, and keywords to exclude 1,371 irrelevant studies. The investigators thoroughly reviewed the full text of 31 citations. Of these, 4 studies were excluded because they lacked extractable effect size data (e.g., hazard ratios, HR) (“lacked target date”), 3 were excluded as they were reviews or meta-analyses, and 3 cross-sectional studies were excluded due to the low level of evidence (lack of temporality). Ultimately, a total of 21 studies were included for quantitative analysis (**Figure 1**).

### Study characteristics and risk of bias

The baseline characteristics of the included studies are presented in **Table 1**. Because of the low level of clinical evidence and poor reliability of cross-sectional studies, we excluded cross-sectional studies and retained only cohort studies for the final meta-analysis. Therefore, all the studies included in our analysis were cohort studies. **Supplementary Table 2** demonstrates that all the included studies had Newcastle-Ottawa Scale (NOS) values ranging from moderate to high. For the meta-analysis, we incorporated a total of twenty-one cohort studies (19–39). Among these, 17 were prospective cohort studies, and 4 were retrospective cohort studies. The combined participant population comprised 2,497,397 individuals, with females representing 55.6 percent of the sample (**Table 1**).

**Table 1:** Baseline characteristics of the included studies in this meta-analysis.

Included studies	Study design	Data source	Region	Sample size	Age (mean, y)	Female (N,%)	Follow-up time(year)	Tumor types	Adjusted confounding factors	NOS
Mandalà-2011	Prospective cohort study	Outcome Research in Oncology Project study	Italy	1,443	51	771 (55.5)	11.8	primary cutaneous melanoma	age and sex	7
Schmidlin-2012	Prospective cohort study	Swiss National Cohort	Switzerland	2,253,378	NA	1,266,744 (56.2)	18	breast cancer; colorectal cancer; lung cancer;prostate cancer	NA	6
Liuu-2020	Prospective cohort study	The geriatric oncology clinic of Poitiers University Hospital	France	433	82.8	181 (42)	1.3	prostate; skin; colorectum; breast;	age, sex, tumor sites and metastatic status	7
Kortsen-2021	Retrospective cohort study	National Danish retrospective penile cancer cohort	Denmark	429	66.33	0 (0)	10	penile cancer	age and cohabiting status	7
Konski-2006	Prospective cohort study	RTOG clinical trials	The United States	1,438	NA	0 (0)	2.3	head and neck cancer	age, marital status, income	6
Allison-2003	Prospective cohort study	the Centre Hospitalier Universitaire	France	101	58.3	7 (6.9)	1.5	head and neck cancer	disease stage, disease site, comorbidity, and treatment modality,FLOT score	7
Eriksson-2014	Prospective cohort study	SMR	Sweden	27,235	62	13,845 (51)	8.4	Cutaneous Malignant Melanoma	age,level of education,living area,period of diagnosis,tumor site,clinical stage,histogenetic type,tumor ulceration,tumor thickness,Clark level of invasion	8
Elovainio-2021	Prospective cohort study	the Finnish Cancer Registry	Finland	46,152	57.1	20,226 (43.8)	5	prostate cancer, lung and tracheal cancer, colorectal cancer, bladder cancer, squamous cell skin cancer, breast cancer, cancer of the corpus uteri	age, social status, rurality, co-morbidity and stage of the cancer	8
Degett-2020	Prospective cohort study	National registries	Denmark	5,310	73	2,595 (48.9)	1	colorectal cancer	sex, age, year of surgery, Charlson Co-morbidity Index score, BMI, smoking status, alcohol consumption, UICC stage and tumor localization	8
Leung-2021	Retrospective cohort study	the Outcomes and Surveillance Integration System	Canada	25,382	NA	12,691 (50)	5	Gastrointestinal cancer,breast cancer,genitourinary cancer,lung cancer	age,sex, disease stage, anxiety, depression, social isolation	6
Kroenke-2020	Prospective cohort study	the Women’s Health Initiative	The United States	1,431	72	1,431 (100)	5.8	Colorectal Cancer	age at diagnosis, time between social assessment and diagnosis, study, race/ethnicity, and stage and grade, site, education, income, family history of colorectal cancer, and comorbidity	7
Wu-2020	Prospective cohort study	The Melbourne Collaborative Cohort Study	Australia	1,421	55	0 (0)	5.3	prostate cancer	tumor Gleason score and stage, and sequentially adjusted for socioeconomic, health- and lifestyle-related confounders	8
Rosskamp-2021	Prospective cohort study	the Belgian Cancer Registry	Belgium	109,591	58.7	60,206 (54.9)	2.6	Colon, rectal, female breast, lung, ovarian, head and neck, pancreatic and stomach cancer	age, sex, stage, subtype, chronic respiratory and cardiovascular comorbidities, diabetes mellitus, and primary treatment scheme.	8
Hjorth-2021	Prospective cohort study	the ProBeCaRe cohort	Denmark	2,616	NA	2,616 (100)	7.2	premenopausal breast	age	7
Frederiksen-2012	Retrospective cohort study	the national Danish lymphoma database	Denmark	6,234	NA	2,813 (45)	NA	non-Hodgkin lymphoma	age, sex, year of operation, and for clustering at the department level;education	8
Raedkjaer-2020	Prospective cohort study	the Danish Sarcoma Registry	Denmark	1,919	NA	861 (45)	14	sarcoma patients	age, gender, comorbidity, and the socioeconomic indicators.	7
Larfors-2016	Prospective cohort study	Swedish CML	Sweden	980	60	441 (45)	4.8	chronic myeloid leukemia	calendar year, age, and WHO performance status at diagnosis	8
Lee-2022	Prospective cohort study	CALGB 89803	The United States and Canada	1,069	NA	474 (44)	7.6	Stage III Colon Cancer	age (continuous), sex (male, female), race (White, other), treatment arm, T-stage (T1-2, T3-4), number of positive nodes (1-3, 4+), performance status (ECOG 0, ECOG 1-2), tumor location (proximal, distal), clinical bowel obstruction or perforation (yes, no), consistent aspirin use (yes, no), insurance status (self-pay/private, Medicare/Medicaid/Military/other), valid FFQ1 (yes, no), household income, time-varying energy intake, BMI, physical activity, Western dietary pattern, prudent dietary pattern (all time-varying variables are continuous)	8
Doyle-2023	Prospective cohort study	The WHEL study	the United States	2,869	50.8	2,869 (100)	7.3	breast cancer	intervention group, age at diagnosis, BMI, cancer stage, estrogen and progesterone status, alcohol consumption, physical activity levels, social support,smoking status, radiotherapy, chemotherapy, menopausal status, and number of comorbidity medications	8
Chen-2024	Prospective cohort study	IRONMAN	International cohort	2,119	71	0 (0)	5	Advanced Prostate Cancer	age at enrollment (continuous), disease state (mHSPC vs. CRPC), continent of enrollment (North America, Europe, and other), self-reported race (white vs. non-white), employment status (not working vs. currently working), smoking status (current non-smoker vs. current smoker), family history of prostate cancer (yes vs. no), and PSA at enrollment (continuous).	8
Asmussen-2025	Retrospective cohort study	population-based health and administrative registries	Denmark	5,847	NA	5,847 (100)	10	breast cancer	age and stage;	7

RTOG, three Radiation Therapy Oncology Group; FLOT, the French version of the Life Orientation Test; SMR, the Swedish Melanoma Register; BMI, body mass index; UICC, Union for International Cancer Control; ProBeCaRe, Predictors of Breast Cancer Recurrence; CML, chronic myeloid leukemia; CALGB, Cancer and Leukemia Group B; WHEL, the Women’s Healthy Eating and Living; NHIS, The National Health Interview Survey; ECOG, Eastern Cooperative Oncology Group; FFQ, food frequency questionnaire; IRONMAN, International Registry for Men with Advanced Prostate Cancer.

### Living alone and all-cause mortality among cancer patients

**Figure 2:**
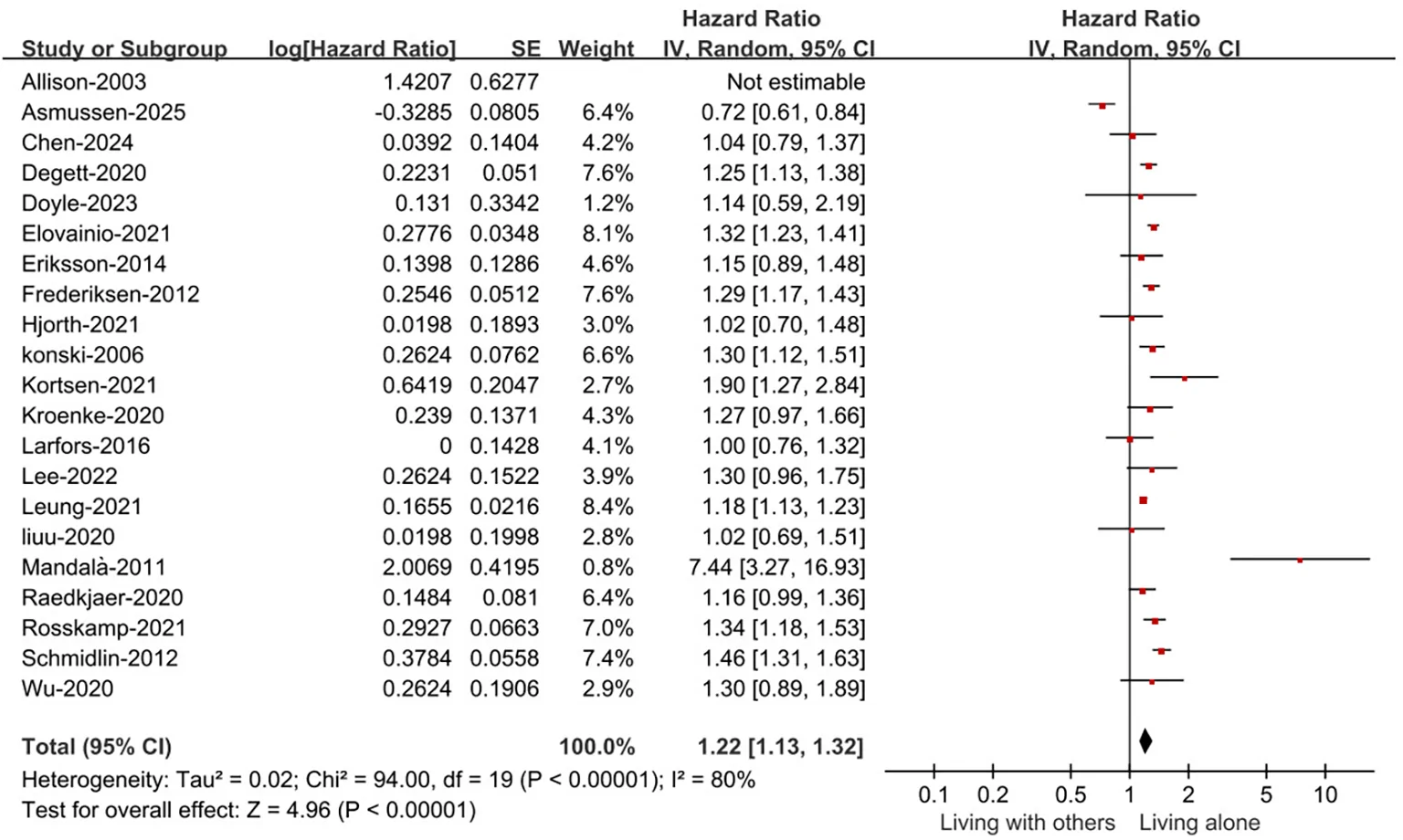
Forest plot for the association of living alone with the risk of all-cause mortality in cancer patients. CI, confidence interval; SE, standard error; IV, inverse of the variance.

All of the included studies reported on the relationship between living alone and mortality risk in cancer patients. Overall, the pooled data imply that living alone is associated with a higher risk of all-cause mortality in cancer patients (HR = 1.22, 95% CI: 1.13–1.32) (**Figure 2**).

### Sex differences between living alone and risk of all-cause mortality

**Figure 3:**
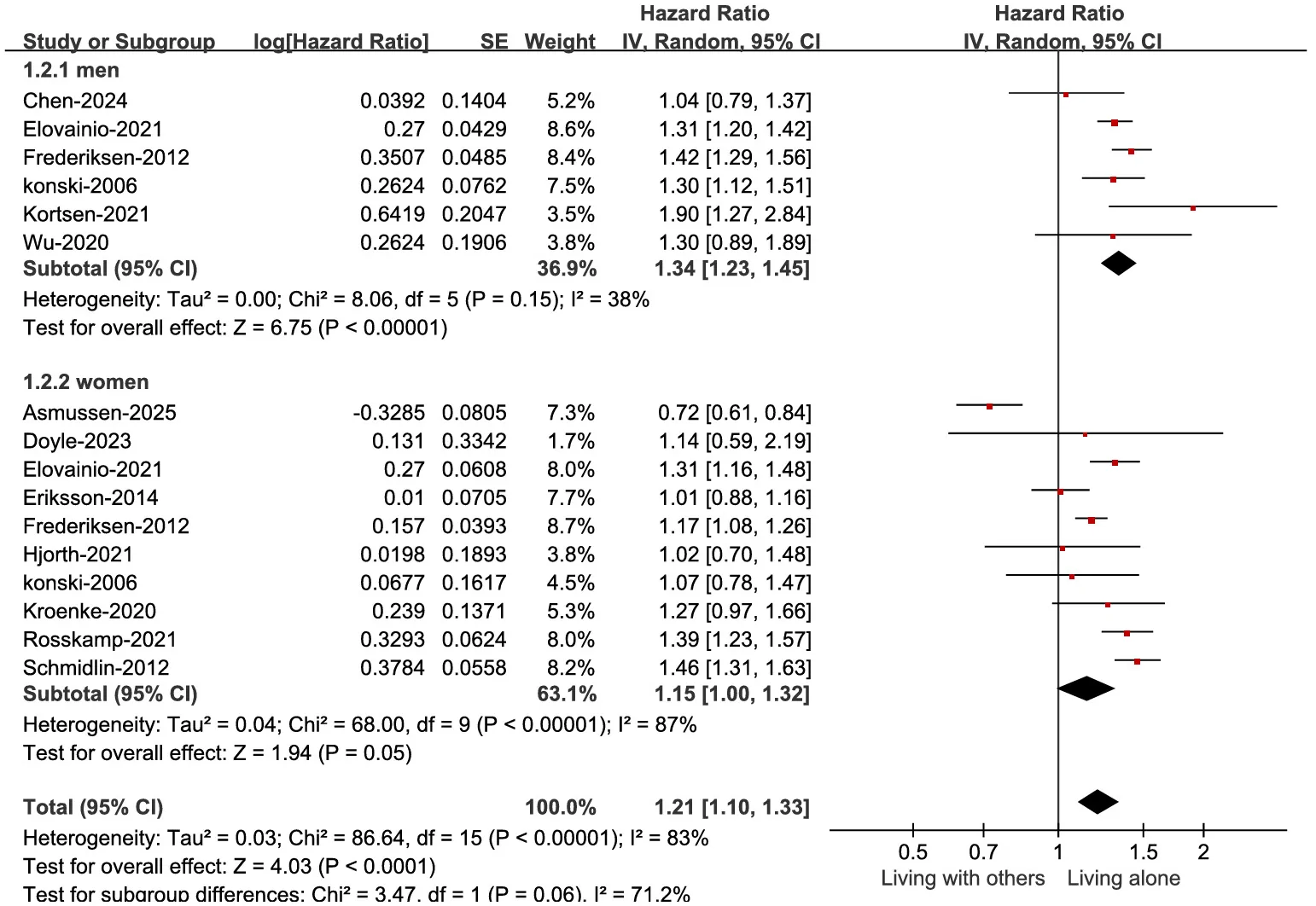
Forest plot for sex differences in the risk of cancer all-cause mortality associated with living alone. CI, confidence interval; SE, standard error; IV, inverse of the variance.

We conducted a subgroup analysis based on sex to explore potential variations in the risk of cancer all-cause mortality among individuals living alone, specifically in males and females with cancer. However, not all of the literature provided HRs and CIs for all genders. In the subgroup analysis, no sex difference in this risk was found between men (HR 1.34, 95% CI 1.23 - 1.45) and women (HR 1.15, 95% CI 1.00 - 1.32) (**Figure 3**).

### Sensitivity analysis of living alone and the risk of cancer mortality

To investigate the sources of heterogeneity observed in the pooled results, we conducted a leave-one-out sensitivity analysis by sequentially excluding each included study (**Supplementary Table 3**). The pooled HRs ranged from 1.21 to 1.24, and the I² values ranged from 64% to 81%, remaining consistently significant across all iterations (all *p* < 0.00001). Notably, the exclusion of Asmussen-2025 reduced the I² from 80% to 64%, suggesting that this study may have contributed to the observed heterogeneity. Overall, the sensitivity analysis confirmed that the association between living alone and increased mortality risk remained stable and robust, with no single study driving the overall estimate.

### Publication bias

In our primary analysis, the funnel plot indicates a slight presence of publication bias, although the results remain robust (**Supplementary Figure 1**). For the sex-specific subgroup analyses, funnel plots also suggested some asymmetry (**Supplementary Figure 2**); however, these findings should be interpreted with caution, as funnel plot asymmetry tests are unreliable when fewer than 10 studies are included in a subgroup. Given the limited number of studies available for individual cancer types, we did not perform funnel plot analyses for tumor-specific subgroups.

## Discussion

In contemporary society, living alone is a common and seemingly inevitable living arrangement that can occur at different life stages and depends on age, sex, education, and employment status. Our study found that cancer patients living alone had a significantly higher risk of all-cause mortality compared to non-living alone cancer patients, with no clear sex difference. Living alone seems inevitable, especially with changes in family patterns and urbanization. The number of older adults living alone is increasing in Asian countries such as South Korea, Japan, and China (2). The population living alone experiences a decrease in social networks and a loss of interpersonal relationships, which may increase the risk of adverse effects on health. Thus, living alone may emerge as an inevitable public health issue.

Previous studies have shown a positive correlation between living alone and greater susceptibility to a variety of physical illnesses such as diabetes and cardiovascular disease (8–10), as well as mental health disorders such as depression and anxiety (40–42). The impact of living alone on cancer patients is also increasingly being recognized. Some studies have shown an association between living alone and the risk of all-cause mortality from cancer, involving tumors such as prostate, breast, lung, trachea, uterus, colon, bladder, and cutaneous melanoma (20, 33, 35). Moreover, among patients with sex specific cancers (such as breast cancer and prostate cancer), there is a sex difference between living alone and cancer death risk. However, there is currently no relevant meta-analysis to clarify this risk. Therefore, we synthesized existing literature to explore the association between living alone and all-cause cancer-related mortality. Our findings suggest that living alone is associated with an increased risk of all-cause mortality in cancer patients. The pooled HR of 1.22 observed in the present meta-analysis can be compared with findings from a systematic review and meta-analysis targeting general community-dwelling adults. Zhao et al. (16) reported that living alone was associated with a 24% increased risk of all-cause mortality (adjusted HR = 1.24, 95%CI 1.17–1.31) in the general adult population. These similar relative risk magnitudes suggest that the proportional increase in mortality associated with living alone is comparable for cancer patients and cancer-free community adults. Importantly, however, cancer patients exhibit markedly higher baseline mortality. As a result, the absolute excess mortality risk related to solitary living arrangements may be larger among individuals with cancer, highlighting the clinical relevance of routinely screening living status in oncology practice. Substantial heterogeneity was observed across the included studies (I² ≈ 80%), which may be attributable to differences in study populations, cancer types, definitions of living alone, and adjustment for confounders. Sensitivity analyses confirmed the robustness of the overall estimate, as no single study drove the pooled result.

Earlier studies have shown sex disparities in the risk of experiencing deleterious consequences from living alone. Men faced significantly higher risks compared to women. Women’s greater self-assurance in managing their lives than men’s may be explained, in part, by disparities in social and educational institutions (43). However, given that we did not find a sex difference in this risk, more research is needed to further clarify whether this difference is real or not, as the literature provides limited data on sex risk.

Due to the limited number of studies for each cancer type (most subgroups containing only 2–3 studies) and the substantial heterogeneity across these studies, we did not perform formal meta-analytic subgroup analyses by cancer type. This decision was further supported by the methodological concern that funnel plots and publication bias tests are unreliable when fewer than 10 studies are included in a subgroup. Future research focusing on specific cancer types, with well-defined populations and standardized measures of living arrangements, will be needed to determine whether the association between living alone and mortality differs across cancer sites.

However, the exact mechanisms by which living alone is associated with an increased risk of all-cause mortality from cancer remain unclear. It is important to distinguish between living alone as a measurable living arrangement and the subjective experiences of social isolation or loneliness, as the latter were not directly assessed in the included studies. Nonetheless, living alone may serve as a proxy for reduced day-to-day social interaction and support. Studies have shown that health-related behaviors and physical fitness may be worse in people who live alone (44, 45). This may have different effects at different stages of the disease process, potentially becoming more pronounced after a cancer diagnosis. Second, living alone may reduce social networks and thus be associated with increased health risks. It has been noted that increased social networks are associated with reduced depressive symptoms (46), increased positive health behaviors (47), and stress-related physiological processes, including potentially improved immune function (48). Third, people who do not live alone may have greater access to social support, health care resources, and social support services (49, 50). Although some studies have suggested sex differences in the association between living alone and mortality for certain sex-specific cancers (i.e., prostate, uterine, and breast tumors), our meta-analysis did not detect a statistically significant overall sex difference, consistent with the limited data available for stratified analyses by both sex and cancer subtype.

### Strengths and limitations

This study has several strengths. First, to our knowledge, this is the first meta-analysis to evaluate the association between living alone and mortality risk in cancer patients using data from cohort studies, and it included a large number of participants across 21 studies. Second, we examined the association from multiple perspectives, including sex, geographic region, and follow-up duration. Third, the robustness of our findings was supported by sensitivity analyses, which confirmed that no single study drove the overall estimate.

The present study has several limitations. First, cross-sectional studies were excluded due to lack of temporality between living-alone status and mortality outcome, which may introduce selection bias. Second, residual confounding exists. Although some studies adjusted for tumor stage, comorbidities, novel anti-cancer therapies, social support and psychosocial factors were inconsistently controlled or measured, which may bias the pooled association. Third, the definition and measurement of “living alone” varied widely across studies (single baseline assessment vs repeated detection, different survey questions), contributing to high heterogeneity; we could not perform targeted sensitivity analyses based on unified definitions. Fourth, stratified analyses or meta-regression by age, income, education and urban-rural residence were unfeasible due to insufficient stratified data from original studies. Besides, we failed to distinguish cancer-specific and non-cancer mortality for further analysis. Finally, most studies were conducted in Europe and North America, limiting the generalizability of our results to Asian, African and South American populations with distinct household structures and social support systems. As all included studies are observational, causal conclusions cannot be drawn. Future standardized multinational studies are required to validate our findings.

## Conclusion

Living alone is associated with a significantly increased risk of all-cause mortality among cancer patients, with no clear sex difference. Further research is needed to determine whether this association differs across cancer types.

## Data Availability

The original contributions presented in the study are included in the article/**Supplementary Material**, Further inquiries can be directed to the corresponding author/s.
