# Supplementary material for: Living alone and mortality risk in cancer patients: a systematic review and meta-analysis
Source: Front Oncol. 2026 Aug 11;16:1866474. doi: 10.3389/fonc.2026.1866474 (PMC13503183; doi:10.3389/fonc.2026.1866474)
Supplement: Supplementary file 1 [file DataSheet1.doc]

***Supplementary Material***

**Supplementary Table 1. The search strategies of this meta-analysis**

|  | **Search terms** | **No.** |
| --- | --- | --- |
| **PubMed** | | |
| #1 | Neoplasms OR Tumor OR Malignancy OR cancer | 3,816,653 |
| #2 | living alone | 6,996 |
| #3 | #1 and #2. | 422 |
| **Embase** | | |
| #1 | Neoplasms | 349,189 |
| #2 | Tumor | 4,415,121 |
| #3 | Malignancy | 424,705 |
| #4 | Cancer | 6,388,035 |
| #5 | #1 OR #2 OR #3 OR #4 | 8,220,939 |
| #6 | living alone | 9,166 |
| #7 | #5 and #6 | 869 |
| **Cochrane Library** | | |
| #1 | Neoplasms OR Tumor OR Malignancy OR cancer | 303,785 |
| #2 | living alone | 4,,551 |
| #3 | #1 and #2 | 568 |
| **Ovid Medline** | | |
| #1 | Neoplasms OR Tumor OR Malignancy OR cancer | 3,266,990 |
| #2 | living alone | 6,834 |
| #3 | #1 and #2 | 411 |

**Supplementary Figure 1.The funnel plots for living alone and the risk of all-cause mortality in cancer patients**

**Supplementary Figure 2.Funnel plots for sex differences in the risk of cancer all-cause mortality associated with living alone.**

**Supplementary Table 2. Quality assessment for the included studies**

| Included studies | Selection (0-4 points) | | | | Comparability (0-2 points) | | Outcome (0-3 points) | | | Total poitns* |
| --- | --- | --- | --- | --- | --- | --- | --- | --- | --- | --- |
|  | Representativeness of Exposed Cohort | Selection of Non-Exposed Cohort | Ascertainment of Exposure | Demonstration That Outcome of Interest Was Not Present at Start of Study | Adjust for the important Risk factors | Adjust for other risk factors | Assessment of outcome | Follow-up length | Loss to follow-up rate |  |
| Mandalà-2011 | * | * | * | * | * |  | * | * |  | 7 |
| Schmidlin-2012 | * | * | * | * |  |  | * | * |  | 6 |
| Liuu-2020 | * | * | * | * | * |  | * | * |  | 7 |
| Kortsen-2021 | * | * | * | * | * |  | * | * |  | 7 |
| Konski-2006 | * | * | * | * |  |  | * | * |  | 6 |
| Allison-2003 | * | * | * | * | * |  | * | * |  | 7 |
| Eriksson-2014 | * | * | * | * | * | * | * | * |  | 8 |
| Elovainio-2021 | * | * | * | * | * | * | * | * |  | 8 |
| Degett-2020 | * | * | * | * | * | * | * | * |  | 8 |
| Leung-2021 | * | * | * |  | * |  | * | * |  | 6 |
| Kroenke-2020 | * | * | * | * | * |  | * | * |  | 7 |
| Wu-2020 | * | * | * | * | * | * | * | * |  | 8 |
| Rosskamp-2021 | * | * | * | * | * | * | * | * |  | 8 |
| Hjorth-2021 | * | * | * | * | * |  | * | * |  | 7 |
| Frederiksen-2012 | * | * | * | * | * | * | * | * |  | 8 |
| Raedkjaer-2020 | * | * | * | * | * | * |  | * |  | 7 |
| Larfors-2016 | * | * | * | * | * | * | * | * |  | 8 |
| Lee-2022 | * | * | * | * | * | * | * | * |  | 8 |
| Doyle-2023 | * | * | * | * | * | * | * | * |  | 8 |
| Chen-2024 | * | * | * | * | * | * | * | * |  | 8 |
| Asmussen-2025 | * | * | * | * |  | * | * | * |  | 7 |

＊The Newcastle-Ottawa Scale (NOS) items, with a total score of 9 points, were used to evaluate the quality of the post-hoc analyses of RCTs and observational study which involve the selection of cohorts (0-4 points), the comparability of cohorts (0-2 points), and the assessment of the outcome (0-3 points)

**Supplementary Table 3. The heterogeneity of the included studies through sensitivity analysis**

| **Excluded study arm** | **HR (95% CI)** | **I^2^ (%)** | **p value** |
| --- | --- | --- | --- |
| Allison-2003 | 1.22 (1.13 - 1.32) | 80 | <0.00001 |
| Degett-2020 | 1.23 (1.13 - 1.34) | 81 | <0.00001 |
| Elovainio-2021 | 1.22 (1.12 - 1.34) | 79 | <0.00001 |
| Eriksson-2014 | 1.23 (1.13 - 1.34) | 81 | <0.00001 |
| Frederiksen-2012 | 1.22 (1.12 - 1.33) | 80 | <0.00001 |
| Hjorth-2021 | 1.23 (1.14 - 1.34) | 80 | <0.00001 |
| konski-2006 | 1.22 (1.12 - 1.33) | 80 | <0.00001 |
| Kortsen-2021 | 1.21 (1.12 - 1.31) | 80 | <0.00001 |
| Kroenke-2020 | 1.23 (1.13 - 1.33) | 81 | <0.00001 |
| Larfors-2016 | 1.24 (1.14 - 1.34) | 80 | <0.00001 |
| Leung-2021 | 1.24 (1.12 - 1.36) | 79 | <0.00001 |
| liuu-2020 | 1.23 (1.14 - 1.34) | 80 | <0.00001 |
| Mandalà-2011 | 1.21 (1.12 - 1.30) | 76 | <0.00001 |
| Raedkjaer-2020 | 1.23 (1.13 - 1.34) | 80 | <0.00001 |
| Rosskamp-2021 | 1.22 (1.12 - 1.33) | 80 | <0.00001 |
| Schmidlin-2012 | 1.21 (1.11 - 1.31) | 78 | <0.00001 |
| Wu-2020 | 1.23 (1.13 - 1.33) | 81 | <0.00001 |
| Lee-2022 | 1.22 (1.13 - 1.33) | 81 | <0.00001 |
| Doyle-2023 | 1.23 (1.13 - 1.33) | 81 | <0.00001 |
| Chen-2024 | 1.24 (1.14 - 1.34) | 80 | <0.00001 |
| Asmussen-2025 | 1.247(1.19 - 1.35) | 64 | <0.00001 |
